# The Influence of Selective Laser Melting (SLM) Process Parameters on In-Vitro Cell Response

**DOI:** 10.3390/ijms19061619

**Published:** 2018-05-30

**Authors:** Bartłomiej Wysocki, Joanna Idaszek, Joanna Zdunek, Krzysztof Rożniatowski, Marcin Pisarek, Akiko Yamamoto, Wojciech Święszkowski

**Affiliations:** 1 Faculty of Materials Science and Engineering, Warsaw University of Technology, Woloska 141, 02-507 Warsaw, Poland; asia.idaszek@gmail.com (J.I.); jzdunek@inmat.pw.edu.pl (J.Z.); krozn@inmat.pw.edu.pl (K.R.); wojciech.swieszkowski@pw.edu.pl (W.Ś.); 2 Materialscare LTD, Zwierzyniecka 10/1, 15-333 Białystok, Poland; 3 Institute of Physical Chemistry of the Polish Academy of Sciences Kasprzaka 44/52, 01-224 Warsaw, Poland; mpisarek@ichf.edu.pl; 4 International Center for Materials Nanoarchitectonics (MANA), National Institute for Materials Science (NIMS), Tsukuba, Ibaraki 305-0044, Japan; yamamoto.akiko@nims.go.jp

**Keywords:** pure titanium, selective laser melting, surface properties, surface chemistry, titanium oxides, cell behavior, internal stresses, chemical treatments, heat treatments

## Abstract

The use of laser 3D printers is very perspective in the fabrication of solid and porous implants made of various polymers, metals, and its alloys. The Selective Laser Melting (SLM) process, in which consolidated powders are fully melted on each layer, gives the possibility of fabrication personalized implants based on the Computer Aid Design (CAD) model. During SLM fabrication on a 3D printer, depending on the system applied, there is a possibility for setting the amount of energy density (J/mm^3^) transferred to the consolidated powders, thus controlling its porosity, contact angle and roughness. In this study, we have controlled energy density in a range 8–45 J/mm^3^ delivered to titanium powder by setting various levels of laser power (25–45 W), exposure time (20–80 µs) and distance between exposure points (20–60 µm). The growing energy density within studied range increased from 63 to 90% and decreased from 31 to 13 µm samples density and Ra parameter, respectively. The surface energy 55–466 mN/m was achieved with contact angles in range 72–128° and 53–105° for water and formamide, respectively. The human mesenchymal stem cells (hMSCs) adhesion after 4 h decreased with increasing energy density delivered during processing within each parameter group. The differences in cells proliferation were clearly seen after a 7-day incubation. We have observed that proliferation was decreasing with increasing density of energy delivered to the samples. This phenomenon was explained by chemical composition of oxide layers affecting surface energy and internal stresses. We have noticed that TiO_2_, which is the main oxide of raw titanium powder, disintegrated during selective laser melting process and oxygen was transferred into metallic titanium. The typical for 3D printed parts post-processing methods such as chemical polishing in hydrofluoric (HF) or hydrofluoric/nitric (HF/HNO_3_) acid solutions and thermal treatments were used to restore surface chemistry of raw powders and improve surface.

## 1. Introduction

Additive manufacturing (AM) enables fabrication of implants with complex shapes and high control over their internal architecture. Moreover, the AM implants can be designed using computed tomography, magnetic nuclear resonance or 3D scans as input data [1,2]. Since humans have unique figures and non-symmetrical skeleton, custom-made implants are emerging as the future of treatment of massive tissue losses which could contribute to the increase in patients’ mental comfort and their faster recovery [3,4]. Moreover, the AM allows to introduce open porosity into the metallic implants, making them lighter and easier to anchor in bone. Additionally, lower stiffness of the porous implants prevents the stress-shielding effect [5]. Another pro is that 3D printers with production process in the powder bed allow for reusing the utilized powder and reduction in waste quantities that would arise during conventional fabrication methods such as Computerized Numerical Control (CNC) cutting. Numerous literature reports show successfully printed dental [6,7,8,9], craniofacial [10,11,12] and orthopedic implants [13,14,15] using selective laser melting (SLM) and electron beam melting (EBM) technologies. The SLM stands out because in enables fabrication of very fine structures with complex internal architectures and overhangs with precision and shape fidelity higher than the EBM [16,17,18]. The latest achievements in SLM is the fabrication of stem of hip endoprosthesis with positive and negative Poisson’s ratios. This approach was designed to improve implant–bone contact, and potentially, implant longevity [19]. It is worth mentioning that all implants in the above-mentioned literature were fabricated with different process parameters and post-processed by different chemical or mechanical methods. This difference in connection with large variation in experimental procedures (e.g., differences in cellular design and test environment) caused varying results of cell growth and make them difficult to compare [20].

The key success factor for 3D printing using laser and electron technologies is an appropriate selection of the process parameters such as beam power, scanning strategy, layer thickness and type of atmosphere used [21,22,23,24,25]. In addition to aforementioned, the overall product quality is dependent also on part orientation in relation to the working platform [26] geometry of the support structures [27] and shape and size of used powders [28,29]. The appropriate combination of all these parameters enables to create an object with expected microstructural, mechanical and geometrical properties [30]. This is a difficult task because each of these parameters has a different effect on 3D printed objects: theoretical density, surface topography, dimensional compatibility with the CAD model or mechanical strength. In general, the increase in energy supplied to the material (J/mm^3^) by increasing the laser power and decreasing the scanning speed causes the porosity decrease [31], but also increases the surface development and reduces dimensional compliance with the CAD model [32]. The increase in laser power makes an increase in the texture of the material, which in turn promotes the anisotropy of mechanical properties [33]. Luckily, one of the greatest advantages of 3D printing is the ability to investigate and optimize various process parameters for new materials in one production cycle. This can be done by producing a series of small elements on the platform with various process parameters such as laser power or scanning speed. In this way, 3D printing parameters were developed or optimized for titanium and its alloys [34] or metal-ceramic composites [35].

Despite the multitude of literature describing the impact of individual parameters on mechanical and functional properties, the influence of manufacturing parameters and post-process chemical treatments on the cell response has still not been investigated. The reported studies focused mainly on surface modification of solid and porous titanium scaffolds. For example, Yavari and coworkers [36] investigated the effect of anodizing parameters and heat treatment of SLM titanium discs and found that combination of both processes improves cell adhesion, proliferation and osteogenic differentiation. Wysocki and coworkers [37] found that chemical polishing of 3D porous titanium scaffolds fabricated by means of SLM yields lowers cell seeding efficiency but improves cell proliferation and scaffolds colonization.

In this paper, we present the effect of various SLM parameters on the material properties of solid titanium discs and response of human mesenchymal stem cells. The obtained results of biological test are discussed in the context of surface parameters (i.e., wettability, free surface energy, surface roughness, thickness of oxide layers and internal stresses in the material) induced by processing with different laser power, distance between scanning points and exposure times. In addition, the post-process chemical treatments necessary to remove the unmelted powder particles were shown to be very beneficial for improving the physicochemical properties of the surface of printed titanium implants and the cellular response.

## 2. Results

### 2.1. Influence of the SLM Process Parameters on the Properties of CP Titanium

**Figure 1:**
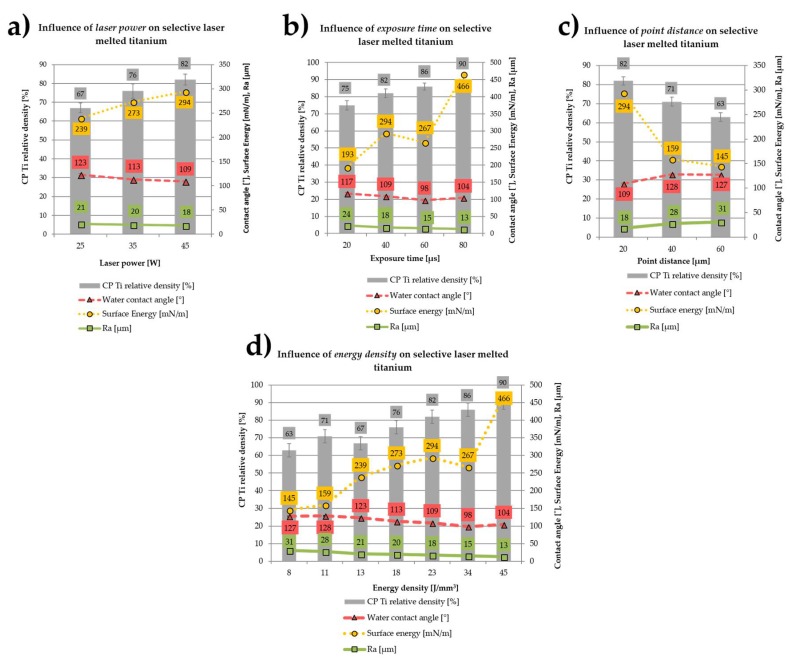
Influence of laser power (**a**), exposure time (**b**), point distance (**c**) and energy density (**d**) on the selective laser melted titanium relative density, surface energy, water contact angle and Ra parameter.

The influence of fabrication parameters (laser power, exposure time and the distance between scanning points) on the selective laser melted titanium density, surface energy, contact angle and Ra parameter is shown in Figure 1a–c. The overall effect of the energy density delivered to the material is shown in Figure 1d. The increase in the laser power in the range of 25–45 W (with a constant exposure time of 40 μs and the distance between the exposure points of 20 μm) yielded 15% and 14% decrease in Ra parameter and water contact angle, respectively (Figure 1a). On the other hand, the enhancement of laser power within the studied range increased the surface energy and material density by almost 23% and 18%, respectively.

Similar to laser power, the increase of the exposure time in the range of 20–80 μs (at a constant power of 45 W and distance between scanning points of 20 μm) resulted in a decrease of Ra parameter by almost 50% (Figure 1b). Increasing exposure time within the range of 20–80 μs reduced the contact angle to up to the exposure time of 60 μs, and then slightly increased at exposure time of 80 µs. The laser exposure time had a great impact on the titanium surface energy which doubled when the laser exposure time was extended within the studied range. The increase in the laser exposure time from 20 to 80 μs also yielded 20% increase in the titanium theoretical density.

A reverse situation was observed when we increased point distance between the laser exposure points (at constant 45 W power and 40 μs exposure time). Enlargement of the spacing increased the Ra parameter by nearly 75% (Figure 1c). By increasing the distance between laser exposure points from 20 to 40 μm, we measured an initial rise in the contact angle which remained unchanged when the point distance was set at 60 μm. Longer distance between exposure points in the range of 20–40 μm yielded surface energy lower by nearly 45% and stabilized at this level. We also measured a reversed correlation between the point distance and the material theoretical density, which dropped by 23% within the studied range.

By increasing the energy density delivered to the material from 8 to 45 J/mm^3^, we changed the density of the discs from 63 to 90% (Figure 1d) and improved their wettability in the range of 8–34 J/mm^3^, with slight reduction of the water contact angle at 45 J/mm^3^. Also, the increase in the total energy density from 8 to 45 J/mm^3^ resulted in a drop of the Ra by nearly 2.5-fold, from 31.2 μm to 12.6 μm. Higher energy density delivered to the material had the reverse effect of the surface energy than the contact angle and Ra parameter. Increase in the energy density delivered to the material within the range of 8–45 J/mm^3^ caused a systematic increase of the surface energy of the material from 145 to 466 mN/m.

### 2.2. Influence of Chemical and Heat Treatments on the Properties of CP Titanium

**Figure 2:**
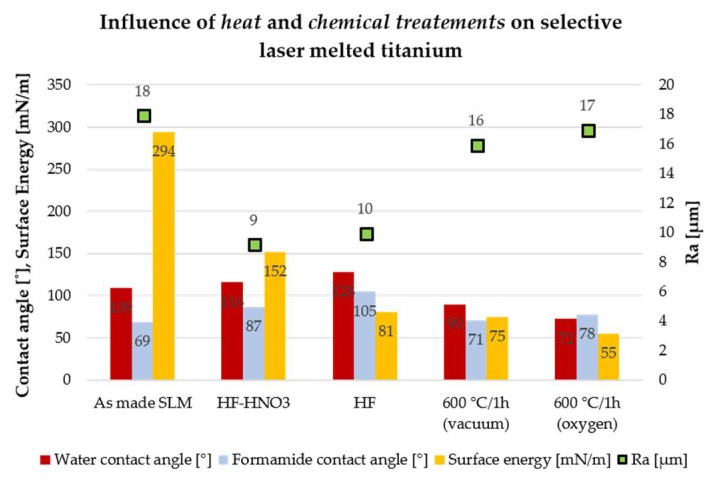
Influence of heat and chemical treatments on Ra parameter, water and formamide contact angle and surface energy.

The influence of chemical and thermal treatments on the Ra parameter, contact angle and surface energy is presented in Figure 2. Samples “as-made” used to determine the effect of chemical and heat treatments on the Ra parameter, contact angle and surface energy were produced at the energy density of 23 J/mm^3^ (45 W laser power, 40 μs exposure time, point distance 20 μm), which was the average value of the energy density delivered to all samples fabricated during the experiment. The water contact angle of SLM fabricated samples increased after chemical treatments in HF acid and the HF/HNO_3_ mixture by respectively 19% and 8%. Thermal treatments caused a drop in the water and formamide contact angle of additively produced objects by 16% and 33%, respectively, for the heat treatment in an oxygen atmosphere and vacuum. The Ra parameter of samples produced decreased by 45–50% after chemical treatments in HF acid and HF/HNO_3_ mixture. Thermal treatments did not change the Ra parameter of the fabricated elements. Both chemical and thermal treatments caused a significant decrease in surface energy-depending on the type of treatment, from 2 times for etching in pure HF to almost 6 times for thermal treatment in the aerobic atmosphere.

### 2.3. Surface Chemistry

**Figure 3:**
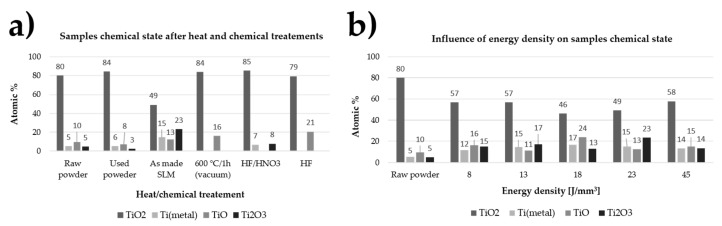
Influence of energy density delivered to the fabricated material (**a**) and heat and chemical treatments (**b**) on samples chemical state.

The effect of SLM process parameters, as well as chemical and thermal treatments on the chemical composition of the top layer of the fabricated samples is presented in Figure 3a,b respectively. Chemical structure of the surface layer of elements produced by the SLM technique varies considerably depending on the energy delivered to the material as well as thermal and chemical treatments. The dominant oxide on the surface of unprocessed powder as well as samples that were fabricated at the energy density of 8 to 45 J/mm^3^ is TiO_2_, but its content decreased from 80% to 50–60% after the laser irradiation process. The chemical composition of the oxide layers were changed after heat and chemical treatments. After the SLM process, the material had on its surface, in addition to TiO_2_, more than 20% Ti_2_O_3_, which did not occur at all after annealing the samples in the furnace under vacuum or after digestion in HF acid alone. This oxide was also detected in samples undergoing chemical treatment in HF/HNO_3_ acid (6 min.) and to a small extent on powders before and after processing.

### 2.4. Residual Stresses

**Figure 4:**
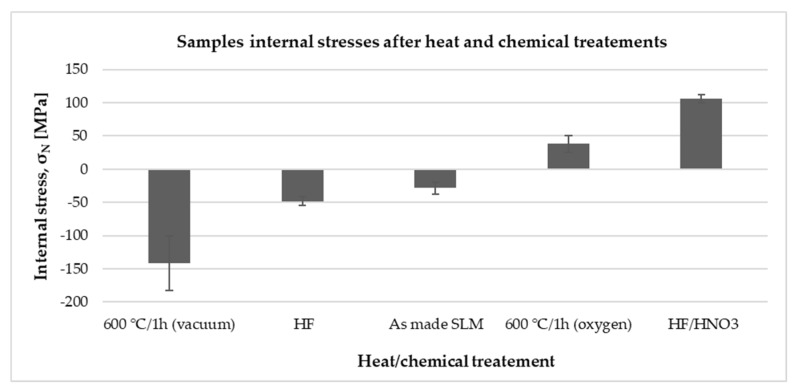
Samples internal stresses after the heat and chemical treatments.

Stress values in the material after the SLM process and subjected to heat and chemical treatments are shown in Figure 4. Depending on the applied treatment, the stresses in the material changed their value and sign. Material after the SLM process had slight compressive stresses, which were significantly increased due to treatment in HF acid and annealing in a vacuum (600 °C). Subjecting the material to strong oxidizing agents, both cleaning in HF/HNO_3_ mixture and heating in the furnace in an oxygen atmosphere also increased the value of stresses in the material but also changed their sign to a positive values.

### 2.5. Cell Behavior

**Figure 5:**
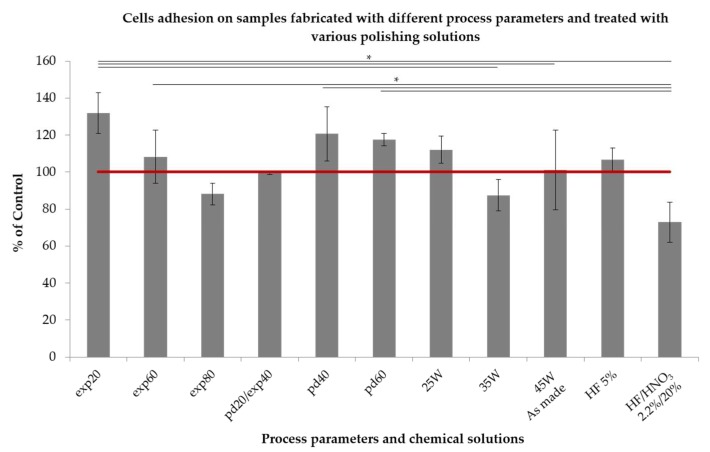
Cells adhesion after 4 h of incubation measured by means of MTS assay. Sample encoded pd20 represent also samples exp40. Samples encoded 45 W were used for chemical polishing, therefore they represent “as-made” reference sample. hMSCs metabolic activity was normalized to the control (100%, red line). *—Significant difference between indicated groups (* *p* < 0.05).

Cell adhesion: The adhesion of the human mesenchymal stem cells (hMSCs) after 4 h of the incubation is shown in Figure 5. The highest metabolic activity of hMSCs was measured on the samples fabricated with the laser exposure time of 20 µs, point distance between exposure points of 40 and 60 µm, and the laser power 25 W. The measured absorbance values equaled 132%, 121%, 118% and 112% of the control for exp20, pd40, pd60 and 25 W, respectively. Within one parameter group, the adhesion decreased with increasing energy density delivered during processing. For example, an increase of exposure time from 20 to 80 μs reduced significantly the adhesion by over 30%. On the other hand, an increase of the point distance between exposure points from 20 to 60 μm yielded adhesion enhanced by 18%. HF treatment did not affect significantly cellular adhesion. On the other hand, chemical polishing with a mixture of HF and HNO_3_ reduced the number of cells adhered to the surface from 101% of the control for the non-treated samples to 73% for the samples treated with a mixture of acids.

**Figure 6:**
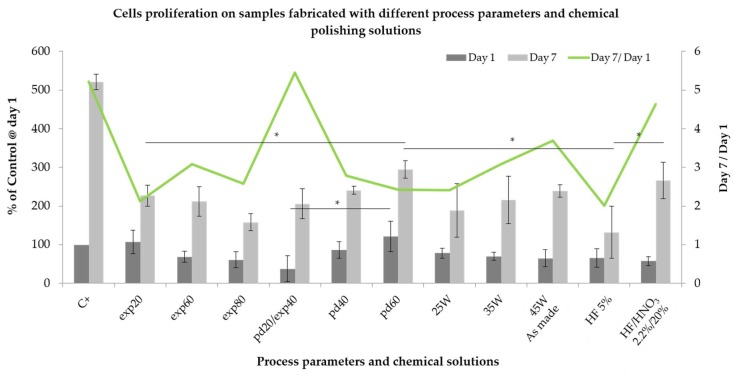
Cells proliferation after 1 and 7 day of incubation measured by means of MTS assay. Sample encoded pd20 represent also samples exp40. Samples encoded 45 W were used for chemical polishing, therefore they represent “as-made”. *—Significant difference between indicated groups (* *p* < 0.05).

Cell proliferation: The potential effect of various process parameters on hMSCs proliferation is shown in Figure 6. At day 1, the pattern of metabolic activity of cells was similar to that observed in the adhesion experiment. Here, the biggest difference among one parameter group was found for point distance between laser exposure points. The measured conversion of MTS increased over 3 times when the point distance was enlarged from 20 to 60 μm. The similarity of the trend observed at day 1 in the proliferation assay can be considered as confirmation of the trend obtained in the adhesion experiment. It may be assumed that after 1 day of incubation, the hMSCs probably did not start to proliferate on the surface of the discs. After 7 days of culture, the highest cell metabolic activity was measured again on the least processed samples for the investigated laser exposure times (i.e., 20 µs) and distances between exposure points (i.e., 20 µm). The opposite effect was observed in the laser power group. In the former two, an increase of exposure time from 20 to 80 μs reduced hMSCs proliferation by approximately 30%, whereas an increase of point distance improved the proliferation by over 40%. Enhancement of the laser power by 20 W yielded over 25% higher MTS conversion. Interestingly, when normalized to the metabolic activity measured at day 1, the biggest increased was observed for the most processed samples, i.e., exp60 and exp80, pd20 and 45 W with the most pronounced proliferation on samples pd20. Absorbance values measured for those samples reached values similar to that in control. With regard to the chemically polished samples, treatment with HF had a negative effect on hMSCs proliferation. After 7 days in culture, cell metabolic activity reached approximately 130%, being the lowest among all tested parameters. Moreover, the increase in MTS conversion between day 1 and 7 was not statistically significant only on the HF-treated samples. On the other hand, we measured the significant increase in absorbance values for the mixture of HF/HNO_3_ acids from 58% of the control at day 1 to 266% at day 7. Such result places this modification among the best with respect to hMSC proliferation.

**Figure 7:**
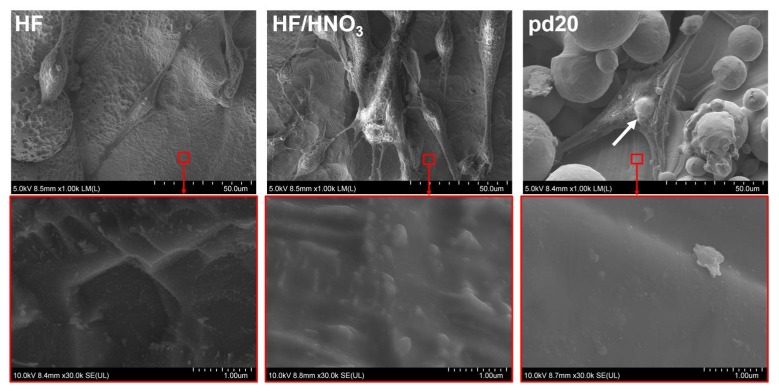
hMSCs under SEM microscope spreading after 1 day of culture on the polished (HF and HF/HNO_3_ solutions) and unpolished (45 W laser power, 40 μs exposure time, 20 μm point distance) titanium discs. Red squares indicate area of the samples which are magnified to show sample topography and are depicted on images below.

**Figure 8:**
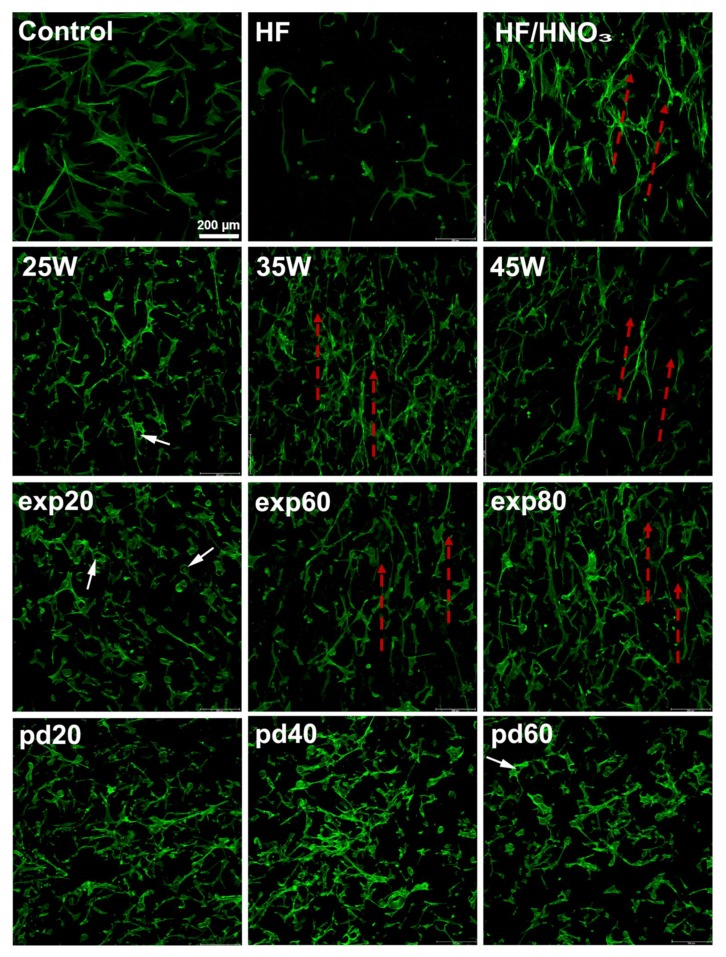
Confocal microscope images of hMSCs spreading after 1 day of culture on the discs fabricated with different laser melting parameters. Cells were stained against F-actin (green). Scale bar of 200 µm.

Cell spreading: Cells on HF/HNO_3_ treated disc acted similarly to those seeded on surfaces fabricated with high energy density. Morphology of cells growing on discs treated with pure HF acid resembled the most that of control. On all surfaces, the hMSC had spindle-like morphology. However, cell growing on the titanium discs were punched by powder particles or grew around them. Unmelted powder particles punching through the hMSC are indicated in Figure 7 and Figure 8 by white arrows. The hMSC appearance differed from that observed on smooth cover glasses. On the polished surfaces (HF and HF/HNO_3_) cells adhered to the disc surfaces while on the unpolished samples they bridged powder particles. This tendency increased with a decrease in the energy density delivered to the samples (more unmelted powder particles). On the other hand, we observed some orientation of cells along paths between powder layers on samples fabricated with increasing energy density exp60/exp80 and 35 W/45 W. Cells spreading along paths between powder layers are shown in Figure 8 by red arrows.

## 3. Discussion

### 3.1. Process Conditions

Implementing a new processing technology, i.e., 3D printing of custom implants, requires prediction of their roughness and physical properties, such as the contact angle and surface energy. If any change in the shape or dimensions of the custom-made implant requires optimization of the process parameters, it must be ensured before the implantation in the living organism that the changed parameters do not critically affect the acceptance of the foreign body. In our work volume based energy density E defined in equation $E=\frac{lp}{\upsilon \cdot h\cdot t}$ (J/mm^3^) (where *lp* is laser power (W), *ν* is scan speed (mm/s), *h* is hatch spacing (mm) and *t* is layer thickness (mm)) was increased by increasing laser power, increasing the exposure time and reducing the distance between exposure points. Our study on the selective laser melting of CP titanium, as well as works on numerous materials such as steel [38], Al–Si [39], Co–Cr [30] and Ti-6Al-4V [40] showed increase of material density with increased energy density delivered during the process. One of the basic quality parameters taken into account in the application of additive manufactured materials is density with value close to the theoretical density of the consolidated material. The lack of pores enclosed in the functional object is primarily to increase their strength by eliminating the areas that can cause crack propagation. To prevent defect formation and obtain during the laser melting process materials with density close to the theoretical values the stability and viscosity of the melt pool should be ensured. The viscosity must be sufficiently low to ensure that the melt can spread properly onto the formerly processed powder layer and in the same time sufficiently high to prevent balling phenomena [41]. The viscosity decreases with increasing deposition temperatures which are achieved with higher energy densities delivered to the material. This result that after exceeding limit value of the energy density material relative density decrease because melt pool became instable [41]. At this point, it is worth to add that the key to success in achieving complex geometry, proper microstructure and high strength of materials by the selective laser melting is not only delivering desired energy density to the consolidated powder, but also the appropriate selection of parameters influencing it such as laser power and exposure time [42].

**Figure A1:**
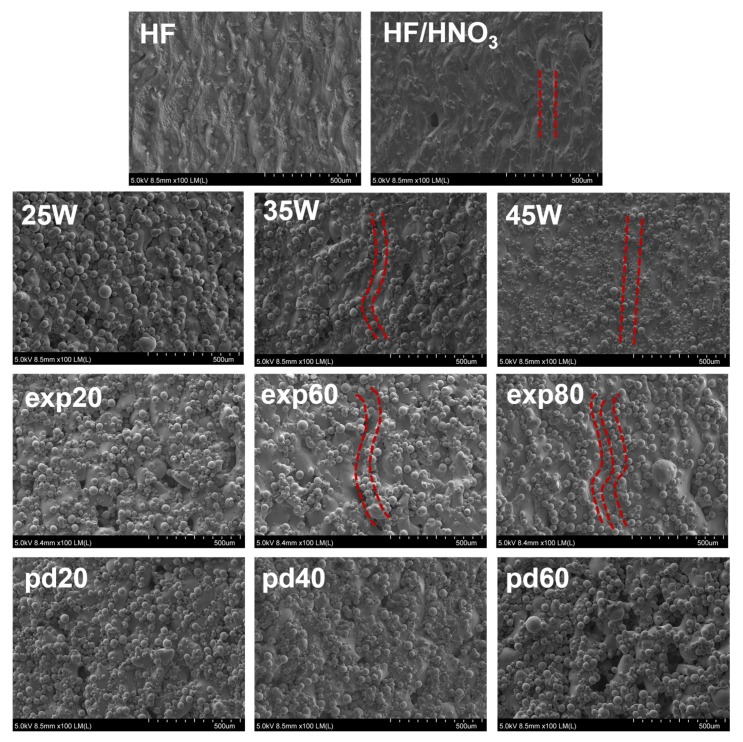
Morphology of discs fabricated from titanium with different SLM process parameters and treated with HF or HF/HNO_3_ acids. Red dashed lines show paths between layers.

The increase of the laser power and exposure time and decreased distance between exposure points caused a decrease in the value of Ra parameter from 31 to 13 µm for samples fabricated with 8 and 45 J/mm^3^, respectively. This effect should be explained by increasing the degree of melting the powder used for consolidation and is presented in Figure A1 (Appendix B). The similar change of Ra parameter was obtained after polishing as-made samples fabricated with 23 J/mm^3^ in HF and HF/HNO_3_ solution acids. The Ra parameter after chemical etching was around 10 µm irrespectively from bath composition. All samples fabricated in our study had relatively high microroughness and should promote osteointegration [43,44,45]. There are scientific reports showing that increasing surface roughness of titanium or titanium alloys in range from 0.3 to 3 µm increase water contact angle to more hydrophobic values above 70°, when surface is covered with rutile-type oxides only [46,47]. Opposite trend of increasing hydrophilicity was observed when surface was covered with mixture of rutile and anatase oxides [47]. On the other hand, Giljean et al. [48] showed that contact angle change of titanium substrates not only depend on the roughness in range 1–20 µm, but also on the effectiveness of the cleaning procedure. It was shown that the contact angle first increases with the roughness parameter, until a threshold value (Ra parameter around 10 µm) from which it levels off (plateau). Although all SLM fabricated discs had high Ra parameter (13–31 µm) and surface energy (145–466 mN/m), their surface was hydrophobic with contact angle values above 98°. We have shown in our previous study similar contact angle values 80–100° obtained for unpolished rough selective laser melted titanium fabricated with energy densities as high as 150 J/mm^3^ [49]. The contact angle of fabricated discs was at least tens of degrees higher than for titanium alloys fabricated conventionally [46,50,51]. Minimizing the contact angle and maximizing the surface energy is recognized as beneficial for obtaining biomaterials that promote cell response [52,53]. The reported in our study increasing surface energy with decreasing water contact angle was in compliance with other authors [54] for all samples fabricated in range 8–23 J/mm^3^. The unexpected decrease in wettability and increase of surface energy for samples fabricated with 34 and 45 J/mm^3^ suggested that other crystalline structure of the oxide films formed on these materials [55,56]. The disadvantage of the metals 3D printing technologies is the necessity of post-processing which should be performed to restore equilibrium microstructure by heat treatments [57] or remove unmelted powder particles by chemical methods [37]. In our study heat and chemical post-processing methods changed values of surface parameters in a different way. Post-process treatments in HF acid or a mixture of HF/HNO_3_ acids reduced the Ra parameter value by removing the unmelted powder particles but caused also a 2–3-fold decrease in the surface energy and increased water contact angle to more hydrophobic values. The reduction of contact angle after chemical polishing, despite the Ra parameter decrease, resulted from the changes of surface nano-topography and stoichiometry of oxides formed on the surfaces. As can be seen in Figure 8, despite the significant removal of titanium particles, the areas on the direct contact of cells with discs had a more developed surface at the nano-scale. The Ra parameter is arithmetical mean deviation of the assessed profile and measured in this study described the micro-roughness of the surface area of 3 mm × 3 mm. Since we used small droplets for measurements of contact angles of water and formamide, the key influence on the obtained values had the nano-topography of the surfaces directly contacting with a liquid droplet. The topography changes of titanium surfaces were not observed under high magnifications above ×30,000 on untreated discs fabricated with different laser parameters. Tendulkar et al. [58] reported intervertebral disc made from titanium wire with Ra around 15 µm and contact angle almost 90° which remained almost unchanged after electro-polishing, while Ra parameter decreased around 3 times. In our study the 4–6-fold decrease in the surface energy of the titanium discs with the simultaneous lack of improvement of parameter Ra resulted from thermal treatments in 600 °C for 1 h. Furthermore annealing changed the contact angle to more hydrophilic values equal 90° and 72° after treatment in vacuum and oxygen atmosphere, respectively. The decrease of surface energy with decreased water contact angle after heat and chemical treatments resulted from cumulative change of oxides stoichiometry and internal stresses. Further alteration of fabricated surfaces to decrease contact angle can be conducted for example by UV irradiation [59].

### 3.2. Surface Chemistry and Residual Stresses

In order to determine the causes of changes in the contact angle and surface energy of samples fabricated with different energy density delivered to the material and subjected to thermal and chemical modifications, we preformed measurements of the oxide layer structure by X-ray Photoelectron Spectroscopy (XPS) and internal stresses applying the X-ray Diffraction XRD method. Regardless of the energy density delivered to the titanium powder, chemical structure of the oxide layers is comparable and all of them include titanium metal as well as TiO_2_, TiO and Ti_2_O_3_. Despite the comparable chemical composition of raw powder and SLM fabricated discs, we observed significant differences between them in the content of oxides. The titanium powder used to fabricate samples by means of the SLM method had nearly 80 at % TiO_2_ on the surface, while samples made of it using the energy in the range of 8–45 J/mm^3^ had only 46 at % up to 57 at % TiO_2_. Melting of the titanium powder by delivering energy to the consolidated powder made a drop of at % TiO_2_ with a simultaneous increase in metallic titanium content from 5.3% to 12–15 at %, respectively for the powder used and the samples made. The reverse dependence is observed for TiO and Ti_2_O_3_, of which at % increases 2–3-fold after the SLM process in relation to the powder used. The increase in at % metallic titanium and a drop of at % TiO_2_ after the SLM process could be caused by the disintegration of oxides during laser irradiation and transfer of oxygen to the metallic titanium causing solid solution strengthening. This hypothesis is probable because literature reports showed the possibility of titanium solid solution strengthening by both conventional powder metallurgy [60] and 3D printing methods [61]. The disintegration of oxides resulting in the change of surface properties could be a reason of unexpected change of surface energy and contact angle for samples fabricated with 34 and 45 J/mm^3^. We hypothesize that by increasing the energy density to 34 J/mm^3^, the surface was enriched in anatase oxides. This led to reduction of the contact angle and altered surface energy [47]. When energy density of 45 J/mm^3^ was delivered to the material the slight increase in contact angle could be connected with increase of rutile TiO_2_ at %. The unusual two fold growth of the surface energy in comparison to other samples could be associated with internal stresses which typically increase with the energy delivered during selective laser melting [62]. Values of the contact angle for samples produced with an energy density of 8–45 J/mm^3^ correlated with the change of at % TiO_2_, which decreased with decreasing contact angle. It is worth mentioning that the titanium powder used during SLM processing had higher at % TiO_2_ than raw one, thus could change properties of fabricated samples in the next manufacturing process. Surface chemistry of both HF and HF/HNO_3_ polished samples changed significantly after chemical post processes. Although the oxide films on SLM fabricated samples were estimated on around 15–35 nm [63], the HF or HF/HNO_3_ treatments removed completely oxides formed during raw powder processing. An increase in TiO_2_ content of about 30 at % on titanium elements fabricated in the SLM process, was obtained by thermal treatments (600 °C/1 h) in a vacuum as well as digestion in HF and HF/HNO_3_ acids. Vacuum processing and pure hydrofluoric acid caused the breakdown of Ti_2_O_3_ oxides with a simultaneous increase in at % TiO. Chemical and heat post processing changed chemical state of surfaces, as well as the value of their internal stresses what should be explained by the stoichiometry of fabricated oxides mostly at % TiO_2_. Altered surface chemistry had decreased surface energy to values two times lower than for SLM as made samples (Table A2). The polished and heat treated surfaces had higher water and formamide contact angle then untreated discs what was connected with TiO_2_ at % increase. The SLM fabricated sample had balanced number of TiO, TiO_2_ and Ti_2_O_3_ oxides and relatively low value of compressive stresses which increased even fivefold when intensive chemical or heat treatments, changing the oxides composition, were involved. hMSC cells were less spread on these surfaces, which suggest that surface chemistry had higher influence on their behavior than other parameters. The changes of oxide types should be further investigated by XRD and TEM methods to define which crystal structure of titanium dioxide (TiO_2_) was presented in selective laser melted titanium.

### 3.3. Cell Behavior

In this study, we investigated the effect of various SLM parameters on hMSCs performance. The parameters were divided into four groups: exposure time, laser point distance, laser power and chemical polishing with two types of acid solutions. In each group, titanium discs with different surface roughness, wettability and surface energy were obtained. It is well known that the aforementioned surface parameters affect the extent and strength of cell adhesion and, by this, their proliferation and differentiation. In the adhesion experiment, a trend could be observed indicating that more cells attached to samples with less energy delivered during processing. Those discs were made of less-melted titanium powder and exhibited higher surface roughness and hydrophobicity and lower surface energy. Cell adhesion to artificial materials depends on adsorption and conformation of proteins present on their surface. Higher protein adsorption is achieved on rough surfaces with moderate hydrophilicity and surface energy and positive surface charge [64,65]. In this study, we measured higher cell adhesion to more hydrophobic but rougher discs. Deligianni and coworkers also observed higher hMSC adhesion to Ti discs polished with SiC metallographic paper, which exhibited higher surface roughness [66]. The authors concluded that the better adhesion resulted from improved adhesion of fibronectin—one of the key proteins mediating anchorage-dependent cell adhesion. This could be confirmed by Khang and co-workers, who found that adsorption of fibronectin is enhanced on rougher surfaces [67]. However, samples produced in this study had roughness two orders of magnitude higher than in the mechanically polished. Therefore, it might be concluded that the cells were mechanically entrapped between unmelted titanium powder particles. This can be confirmed by reduced adhesion on HF/HNO_3_ polished samples, which exhibited approximately 40% lower roughness. This corresponded to adhesion lower by almost 30%. However, cell adhesion to HF-treated was samples similar to that in control. This suggests that also internal stress and oxides stoichiometry could influence cell behavior. Samples treated with HF/HNO_3_ exhibited the highest positive internal stress, whereas the stress in the HF-treated samples was negative and slightly higher than in the non-treated. Furthermore polished in HF/HNO_3_ solution samples had higher at % TiO_2_, and no TiO in comparison to HF-treated ones.

HMSC adhesion had a direct effect on cell proliferation. After 7 days of culture, we measured cell metabolic activity approximately two times higher than at day 1 for the majority of the tested parameters. This can be attributed to higher initial cell number rather than the increased rate of cell divisions. However, we measured the increase in proliferation rate similar to that in control for samples pd20 and treated with a mixture of HF/HNO_3_ (approximately five-time increase). Also, cell morphology on the later surface resembled more morphology of cells cultured on cover glasses. This could be a result of the removal of unmelted titanium powder which led to the smoother surface without particle obstacles. Moreover, HF/HNO_3_ treated samples had approximately 50% higher surface energy than the HF-treated. Similar dependence of cell morphology and proliferation rate and surface roughness was observed by Kunzler and coworkers on surface roughness gradients [68].

## 4. Materials and Methods

### 4.1. Fabrication of Samples

**Figure 9:**
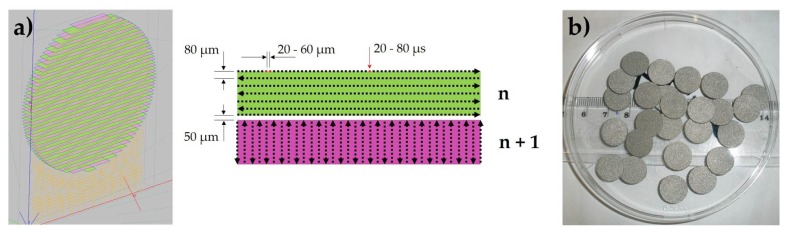
Samples orientation and scanning strategy (**a**), “as-made” samples after cleaning (**b**).

The sets of disks with dimensions of 12 mm diameter and 2 mm thickness were fabricated from CP titanium (Starbond Ti4, Scheftner Dental Alloys GmbH, Mainz, Germany) powder dedicated for dental applications on Realizer SLM50 (Realizer GmbH, Borchen, Germany) 3D printer. According to the manufacturer’s data, the Ti4 powder had a diameter of 10–45 µm, met titanium Grade 4 requirements and its purity was minimal 98.95 wt %. (max impurities: 0.5% Fe, 0.4% O, 0.08% C, 0.05% N and 0.125% H). The SLM process was conducted in an environmental chamber using 0.4 vol % of oxygen. The energy density used for powder consolidation was in the range from 8 to 45 J/mm^3^. The energy density values were changed by laser power, exposure time and point distance in the range 25–45 W (1000–1800 mA), 20–80 μs, and 20–60 µm respectively. The set of samples fabricated with a mean value of energy density (23 J/mm^3^) was used as a reference point for heat and chemical treatments influence and marked “as-made” (Figure 9b). For each set of the process parameters or the post processing method 15 disc samples were fabricated. The applied process parameters are shown in Figure 9a and summarized in Table 1. The detailed set of parameters is described in Table A1 (Appendix A). After fabrication and removal of support structures, the unsintered powder was removed by washing the samples several times in isopropyl alcohol and then distilled water using an ultrasonic cleaner. After ultrasonic cleaning, the density of 10 samples for each fabricated set of parameters or post-processing treatment, was determined by Archimedes method using a RADWAG WPS 510/C/2 (Radwag, Radom, Poland) balance. The three-dimensional surface roughness was measured on the round surface of the solid plates using a Veeco Wyko NT9300 optical profilometer (Brucker, Billerica, MA, USA) in the VSI (Vertical Scanning Interferometry) mode. The area of 3 mm × 3 mm was measured with resolution of 2.5 µm^3^. The three-dimensional surface roughness measurements were made on one representative sample made with selected manufacturing parameters and after heat and chemical treatments. Presented value of Ra parameter is average value for both sides.

### 4.2. Chemical and Heat Treatments

Chemical polishing and heat treatments were performed for a set of samples fabricated with a mean value of energy density delivered for all samples in this study (23 J/mm^3^) and labeled “as-made”. Chemical polishing was performed in ultrasonic cleaner in the hydrofluoric acid solution (5%–3 min.) and in a mixture of hydrofluoric and nitric acids (2.2/20% HF/HNO_3_–6 min.)*,* which was chosen based on our previous study on titanium scaffolds for bone tissue engineering [37,49]. As-made samples were annealed at 600 °C for 1 h in an air atmosphere or vacuum and then cooled with a furnace.

### 4.3. Contact Angle and Surface Energy

Contact angle and surface energy measurements were made using the Contact Angle System OCA (DataPhysics, Filderstadt, Germany). The 15 measurements on the 15 samples were performed on each material type (manufacturing parameter and thermal or chemical treatment) using 2 mediums (distilled water and formamide). We have tried to perform measurements also on diiodomethane but it was spreading immediately after putting its droplet on discs surface. During measurements, 1 µL of each medium was placed on the samples, photographed and analyzed using software delivered with the goniometer. On the basis of 15 measurements, the software provided by the goniometer manufacturer determined surface energy using the Owens–Wendt method. The contact angle values for water and formamide are presented in Appendix A (Table A2).

### 4.4. X-ray Photoelectron Spectroscopy (XPS)

The chemical composition of the Ti powders and disc samples were examined using X-ray photoelectron spectroscopy, with a Microlab 350 spectrometer (Thermo Electron, Waltham, MA, USA). The measurements were made on one representative sample made with selected manufacturing parameters and after heat and chemical treatments. The scanning area of the X-ray beam was 2 mm × 5 mm (0.1 cm^2^), what gave us the average information from each of the investigated surfaces. The XPS spectra were excited using AlKα (hν = 1486.6 eV) radiation as a source. The high-resolution spectra were recorded using 40 eV pass energy at 0.1 eV step. A linear or Shirley background subtraction was made to obtain the XPS signal intensity. The peaks were fitted using an asymmetric Gaussian/Lorentzian mixed function. The measured binding energies were corrected in reference to the energy of C 1 s at 285 eV. Advantage-based data system software (Version 5.97) was used for data processing.

### 4.5. Determination of Residual Stresses

The residual stresses were measured using the Bruker D8 Discover X-ray diffractometer by PSD VATEC position-sensitive detector (Brucker, Billerica, MA, USA) with a point beam collimated to approximately 1.5 mm Co Kα (1.79 Å) radiation. The recording conditions were: 40 kV voltage, 40 mA current, Δ2Θ 0.03 step and 200 s/step counting time to measure the stress. Measurements were provided by sin^2^Ψ method which is considered a non-destructive method among many stress determination methods. The sin^2^Ψ method is based on plotting changes in interplanar distance as a function of the sample inclination by angle Ψ (sample inclination was in the range of 0–60°, while ΔΨ = 10°). The measurements were made on one representative sample fabricated with selected manufacturing parameters and after heat and chemical treatments.

### 4.6. Cell Culture

Sample preparation: Samples were sterilized in 70% ethanol for 1 h and washed 3 times with PBS. Subsequently, the samples were incubated overnight in expansion medium consisting of α-MEM (Gibco, Paisley, UK) supplemented with 10% fetal bovine serum (FBS, Biowest, South America origin, Riverside, MO, USA), 1% of antibiotics (10,000 U/mL penicillin and 10 µg/mL streptomycin; Gibco, Grand Island, NY, USA) and 1 ng/mL human basic fibroblast growth factor (Sigma-Aldrich, Israel) in CO_2_ atmosphere to ensure protein equilibrium.

Cells: Normal Human Bone Marrow-Derived Mesenchymal Stem Cells (hMSCs) were purchased from Lonza (Walkersville, MD, USA). The cells were isolated from bone marrow of 41 years old female. The cells used in the experiments were from passage 4.

Seeding: hMSCs were seeded at a density of 2 × 10^4^ per disc (adhesion experiment) and 5 × 10^3^ per disc (proliferation experiment) and incubated in expansion for 4 h (adhesion) and 1 and 7 days (proliferation). A 10 μL droplet containing the desired cell number was placed on the discs and the cells were allowed to attach for 30 mins. Then, the warm medium was slowly added.

Cell viability: The MTS assay (CellTiter 96^®^ AQueous One Solution Cell Proliferation Assay; Promega, Madison, WI, USA) was carried out to determine the change of viable cell number. At predetermined time points, the discs incubated with cells were washed with α-MEM w/o FBS and placed in a new 24-well plate containing 400 µL of α-MEM w/o FBS. Then, 80 µL of MTS was added to each well and incubated for 2.5 h. After the incubation, 100 μL aliquots of the supernatant were transferred into new wells of 96-well plate and the absorbance was measured at λ = 490 nm.

Statistics: The data are expressed as a mean ± standard deviation (SD). The results were evaluated statistically by means of post hoc one way ANOVA with a Tukey–Kramer pair-wise comparison test (KyPlot 2.0 beta 15 freeware software, KyensLab Inc., available online: www.kyenslab.com).

### 4.7. Cell Observations

Confocal and scanning electron microscopy: Cells cultured for 1 and 7 days were fixed with 4% paraformaldehyde (confocal microscopy) or 3% glutaraldehyde solution (scanning electron microscopy, SEM). For confocal microscopy, the F-actin (cell cytoskeleton) was stained with Alexa Fluor 488 Phalloidin (Molecular Probes, Eugene, OR, USA) and cell nuclei were stained with Draq5 dye (Thermo Scientific, Rockford, IL, USA) and visualized at 488 and 633 nm wavelength, respectively, using Leica TCS SP8 confocal microscope. For scanning electron microscopy, the fixed cells were washed 3× with deionized water and dehydrated with ethanol gradient (50%, 70%, 90% and 99%) and hexamethyldisilazane treatment (Fluka, Buchs, Germany). The cells were visualized at an acceleration voltage of 5–10 keV in SE mode at various magnifications using Hitachi SU8000 microscope (Hitachi Ltd., Tokyo, Japan).

## 5. Conclusions

In this study, we investigated the effect of various SLM parameters on the material properties of solid titanium discs and correlated them with response of human mesenchymal stem cells. Our work showed that surface properties (roughness, wettability, surface energy) of the additive manufactured materials could be controlled by the amount of energy density (J/mm^3^) delivered to the material by various process parameters (laser power, exposure time, point distance between exposure points). The highest theoretical density could be achieved by delivery of the highest energy density. Therefore, high laser power, high exposure time and low point distance are desirable from material point of view (e.g., lower closed porosity of the produced structures). However, they lead to reduced cell adhesion and proliferation. Surface properties and oxide layer composition could be altered by chemical polishing in HF or HF/HNO_3_ acids solutions and heat treatments in both vacuum and oxygen atmosphere. Moreover, the chemical polishing with HF/HNO_3_ acid solution improved cell hMSC proliferation rate. To conclude, SLM implants should be fabricated with high energy densities and chemically polished to obtain high-quality implants with improved hMSC performance.

## 6. Patents

The polishing procedure of selective laser melted titanium by HF/HNO_3_ solutions is a part of patent-pending method of producing objects by additive manufacturing technologies. The patent application priority date is 14 November 2017.

## Figures and Tables

**Table 1:** The process parameters used for samples fabrication.

Parameter	Abbreviation	Value
Laser current (mA)	lc	1000–1800
Laser power (W)	lp	25–45
Exposure time (µs)	exp	20–80
Point distance (µm)	pd	20–60
Hatch spacing (µm)	h	80
Scanning speed (mm/s)	υ	250 ^a^–1500 ^b^
Layer thickness (µm)	t	50
Energy density (J/mm^3^)	E	8 ^b^–45 ^a^
Model size (mm)	S	φ = 12, h = 2

^a^ exposure time 80 µs and point distance 20 µm; ^b^ exposure time 40 µs and point distance 60 µm. Tables with a detailed description of parameters used for samples fabrication are included in Appendix A.

## Appendix A

**Table A1:** Selective Laser Melting (SLM) process parameters used for titanium disc fabrication.

Laser Power lp (W)	Exposure Time exp (µs)	Point Distance pd (µm)	Scanning Speed ν (mm/s)	Energy Density (J/mm^3^)
Increasing laser power (lp)				
25	40	20	500	12.50
35	40	20	500	17.50
45	40	20	500	22.50
Increasing exposure time (exp)				
45	20	20	1000	11.25
45	40	20	500	22.50
45	60	20	333	33.75
45	80	20	250	45.00
Increasing point distance (pd)				
45	40	20	500	22.50
45	40	40	1000	11.25
45	40	60	1500	7.50

The raw highlighted gray is a reference point for all changed process parameters.

**Table A2:** Water and formamide contact angle and calculated surface energy.

Sample	1	2	3	4	5	6	7	8	9	10	11	12	13	14	15	Contact Angle (°)	σ (°)	Surface Energy (mN/m)	Dispersive Energy (mN/m)	Polar Energy (mN/m)	ch2/n (mN/m)
HF-HNO3|water	119	117	115	117	119	115	117	114	116	118	120	115	114	114	118	116	2	152	123	28	4.36 × 10^−15^
HF-HNO3|formamide	87	88	88	89	86	89	87	83	86	83	87	85	87	90	91	87	2				
HF|water	130	127	127	129	129	126	127	129	131	126	129	125	126	128	130	128	2	81	65	16	5.88 × 10^−15^
HF|formamide	107	103	107	104	104	103	106	105	107	104	105	104	104	104	105	105	1				
600 C (oxygen)|water	76	70	75	73	69	68	67	76	74	72	73	74	73	76	70	72	3	55	54	1	8.60 × 10^−15^
600 C (oxygen)|formamide	70	80	90	89	84	79	90	92	67	81	62	61	63	92	74	78	11				
600 C (vacuum)|water	89	88	91	87	87	89	90	91	91	91	94	90	88	93	88	90	2	75	4	71	4.17 × 10^1^
600 C (vacuum)|formamide	74	72	71	68	69	69	71	71	74	73	70	68	68	74	68	71	2				
25 W|water	111	113	115	113	117	111	113	112	116	114	116	116	112	114	114	114	2	239	189	50	1.21 × 10^−14^
25 W|formamide	77	79	80	79	80	78	78	80	78	76	78	78	75	73	78	78	2				
35 W|water	121	120	123	120	122	121	123	126	123	124	126	124	126	123	124	123	2	273	205	68	2.43 × 10^−14^
35 W|formamide	86	84	83	80	81	84	87	86	88	82	88	87	85	84	81	85	3				
45 W (As made)|water	109	107	108	110	110	109	107	107	110	109	109	110	107	111	108	109	1	294	234	61	1.42 × 10^−14^
45 W (As made)|formamide	71	67	66	69	68	69	71	67	69	70	68	73	72	65	69	69	2				
exp20|water	113	117	120	115	112	114	120	121	121	117	115	119	120	121	113	117	3	193	153	40	5.63 × 10^−15^
exp20|formamide	83	86	84	89	83	83	90	85	81	85	89	82	82	86	80	84	3				
exp40|water	109	107	108	110	110	109	107	107	110	109	109	110	107	111	108	109	1	294	234	61	1.42 × 10^−14^
exp40|formamide	71	67	66	69	68	69	71	67	69	70	68	73	72	65	69	69	2				
exp60|water	98	97	96	98	97	99	98	99	99	100	98	100	97	98	98	98	1	267	224	43	8.44 × 10^−15^
exp60|formamide	58	57	58	57	59	57	60	60	58	58	58	58	59	59	61	58	1				
exp80|water	105	102	106	105	105	104	104	103	105	108	102	102	104	103	108	104	2	466	362	104	1.22 × 10^−14^
exp80|formamide	50	55	54	54	50	55	51	55	55	50	53	55	52	51	53	53	2				
pd20|water	109	107	108	110	110	109	107	107	110	109	109	110	107	111	108	109	1	294	234	61	1.42 × 10^−14^
pd20|formamide	71	67	66	69	68	69	71	67	69	70	68	73	72	65	69	69	2				
pd40|water	126	128	129	132	131	127	132	126	127	131	127	130	126	128	127	128	2	159	122	38	1.29 × 10^−14^
pd40|formamide	100	99	102	101	100	97	99	96	96	99	103	95	98	97	98	99	2				
pd60|water	126	129	126	126	128	129	130	127	128	127	127	126	130	130	126	128	1	145	111	34	9.19 × 10^−15^
pd60|formamide	99	94	94	95	97	97	98	97	96	97	99	98	96	97	96	97	1				

The raw highlighted gray is a reference point for all changed process parameters.
